# Interplay between ESKAPE Pathogens and Immunity in Skin Infections: An Overview of the Major Determinants of Virulence and Antibiotic Resistance

**DOI:** 10.3390/pathogens10020148

**Published:** 2021-02-02

**Authors:** Gustavo Henrique Rodrigues Vale de Macedo, Gabrielle Damasceno Evangelista Costa, Elane Rodrigues Oliveira, Glauciane Viera Damasceno, Juliana Silva Pereira Mendonça, Lucas dos Santos Silva, Vitor Lopes Chagas, José Manuel Noguera Bazán, Amanda Silva dos Santos Aliança, Rita de Cássia Mendonça de Miranda, Adrielle Zagmignan, Andrea de Souza Monteiro, Luís Cláudio Nascimento da Silva

**Affiliations:** 1 Programa de Pós-graduação em Biologia Microbiana, Universidade CEUMA, 65075-120 São Luís, Brazil; gustavo.macedo.7@hotmail.com (G.H.R.V.d.M.); gabrielledamasceno.nutri@gmail.com (G.D.E.C.); julianasmendonca2@gmail.com (J.S.P.M.); rita.miranda@ceuma.br (R.d.C.M.d.M.); andreasmont@gmail.com (A.d.S.M.); 2 Laboratório de Patogenicidade Microbiana, Universidade CEUMA, 65075-120 São Luís, Brazil; elaneroliveira5343@gmail.com (E.R.O.); glauciadamasceno5@gmail.com (G.V.D.); ls.luscas@gmail.com (L.d.S.S.); vitorlopes.ch@gmail.com (V.L.C.); adriellyzagmignan@hotmail.com (A.Z.); 3 Programa de Pós-graduação em Odontologia, Universidade CEUMA, 65075-120 São Luís, Brazil; jmnbazan@hotmail.com; 4 Programa de Pós-graduação em Gestão de Programas e Serviços de Saúde, Universidade CEUMA, 65075-120 São Luís, Brazil; amanda_alianca@yahoo.com.br; 5 Programa de Pós-graduação em Meio Ambiente, Universidade CEUMA, 65075-120 São Luís, Brazil; 6 Laboratório de Microbiologia Aplicada, Universidade CEUMA, 65075-120 São Luís, Brazil

**Keywords:** skin infections, chronic wounds, hypervirulent phenotypes, multidrug resistance

## Abstract

The skin is the largest organ in the human body, acting as a physical and immunological barrier against pathogenic microorganisms. The cutaneous lesions constitute a gateway for microbial contamination that can lead to chronic wounds and other invasive infections. Chronic wounds are considered as serious public health problems due the related social, psychological and economic consequences. The group of bacteria known as ESKAPE (*Enterococcus faecium*, *Staphylococcus aureus*, *Klebsiella pneumoniae*, *Acinetobacter baumannii*, *Pseudomonas aeruginosa* and *Enterobacter* sp.) are among the most prevalent bacteria in cutaneous infections. These pathogens have a high level of incidence in hospital environments and several strains present phenotypes of multidrug resistance. In this review, we discuss some important aspects of skin immunology and the involvement of ESKAPE in wound infections. First, we introduce some fundamental aspects of skin physiology and immunology related to cutaneous infections. Following this, the major virulence factors involved in colonization and tissue damage are highlighted, as well as the most frequently detected antimicrobial resistance genes. ESKAPE pathogens express several virulence determinants that overcome the skin’s physical and immunological barriers, enabling them to cause severe wound infections. The high ability these bacteria to acquire resistance is alarming, particularly in the hospital settings where immunocompromised individuals are exposed to these pathogens. Knowledge about the virulence and resistance markers of these species is important in order to develop new strategies to detect and treat their associated infections.

## 1. Introduction

Skin wounds are considered a serious public health problem, resulting in social, psychological and economic consequences [1,2]. Since wounds impair the anatomical continuity of the skin, they substantially increase the risk of microbial contamination, as the lesions constitute a gateway for microorganisms [3,4,5]. In fact, wounds induced by prolonged hospitalizations and surgical interventions have a strong association with healthcare-related infections [6,7].

Once the tissue integrity is impaired, a cascade of biochemical reactions, known as the healing process, is activated to repair the damage [8,9,10]. The healing pathway consists of distinct and overlapping phases comprising homeostasis, inflammation, proliferation, re-epithelialization and tissue remodeling [9,11]. The presence of pathogenic microorganisms extends the inflammation period which is characterized by the exacerbated release of inflammatory mediators in response to bacterial persistence, closely associated to biofilm formation [12,13,14].

**Figure 1:**
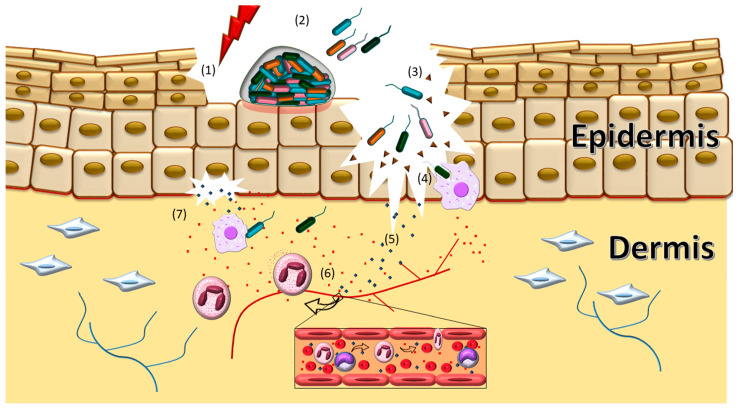
A schematic view of bacterial skin infection, derived from a loss in epidermis integrity. (1) An injury provokes a skin lesion that constitutes a gateway for microbial contamination. (2) Bacteria colonize the skin and produce a biofilm. (3) Bacteria secrete toxins that extend the tissue degradation, reaching dermis layer. (4) Resident immune cells recognize the bacteria and secrete immune mediators to neutralize the pathogens and recruit other immune cells. (5) Cell debris (damage-associated molecular patterns (DAMPS) and lipid mediators) activate immune cells and serve as chemoattractors. (6) Blood leukocytes are recruited to combat the pathogens. (7) Effector substances released by immune cells also promote tissue damage and amplify the inflammation.

Moreover, the cytotoxic action of bacterial virulence determinants results in cell damage and this may amplify the inflammation [15,16,17,18]. The prolongation of the inflammatory phase results in an impairment of the healing process [12,14,19]. In this sense, microbial infections are highlighted as the most important causes of chronic wounds and are usually associated with biofilm formation, which are notoriously recalcitrant to conventional antibiotics [20,21]. The class of microorganisms known as ESKAPE (*Enterococcus faecium*, *Staphylococcus aureus*, *Klebsiella pneumoniae*, *Acinetobacter baumannii*, *Pseudomonas aeruginosa* and *Enterobacter* sp.) are among the most prevalent bacteria in cutaneous infections [22,23,24]. The dynamics of bacterial infection of the skin are illustrated in Figure 1.

Despite the development of several antimicrobial formulations (containing silver derivatives, mupirocin, fusidic acid, mafenide, gentamicin, bacitracin, neomycin and polymyxin B), the treatment of ESKAPE-related skin infections is a huge challenge [25,26,27]. This scenario is due the ability of ESKAPE bacteria to acquire profiles of multidrug-resistance (MDR), extensive drug-resistance (XDR) and pandrug-resistance (PDR) [18,28,29]. Indeed, resistance determinants and plasmids mediating resistance towards topical antibiotics such as mupirocin, fusidic acid and neomycin [30,31,32] and silver have been detected in clinical isolates of ESKAPE bacteria [33,34].

This descriptive review aims to discuss the involvement of ESKAPE pathogens in wound infections, highlighting the major virulence factors involved in colonization and tissue damage and the most frequently detected antimicrobial resistance genes. We also provide an overview of skin physiology and the participation of resident cells and professional immune cells in pathogen detection and the healing process.

## 2. Fundamentals of Skin Physiology and Immunology

The skin constitutes a physical barrier formed by juxtaposed cells that cover the whole body and protect it from environmental variations and traumas [35,36]. The current conception describes the skin as an organ actively involved with the metabolism of macromolecules, and as part of the immune, nervous and endocrine systems. Two distinct and tightly joined layers make up the skin: the epidermis (more superficial) and the dermis (deeper). A third layer, called the hypodermis, is located deeper and consists mainly of adipose tissue [37,38].

The proper functioning of the skin requires close communication and collaboration between various cell types including the stromal cells (keratinocytes, fibroblasts, endothelial cells and adipocytes) as well as those derived from bone marrow (dendritic cells, macrophages, natural killer cells, mast cells, T cells and others) [39,40]. Therefore, this complex organ has a variety of resident cells that play critical roles in detecting invasive organisms (or preventing infections) [41,42].

Molecular signals called damage-associated molecular patterns (examples of DAMPs include ATP, nucleic acids and HMGB1 (high mobility group box 1 protein)) are released from damaged cells. DAMPs can be detected through molecular pattern receptors present in the skin-resident cells [43,44,45,46,47]. These receptors can also identify pathogen-associated molecular patterns (PAMPs), such as peptidoglycans [41,48,49]. The main structures involved in the recognition of DAMPs and PAMPs are the so-called Toll-type receptors. The detection of these receptors by DAMPs and PAMPs activates several mechanisms resulting in the release of pro-inflammatory mediators (such as cytokines, nitric oxide) and the secretion of antimicrobial compounds [48,50,51,52].

Altogether, the skin cells and immune cells form the concept of the skin-associated lymphoid tissue (SALT), which acts as a tertiary lymphoid organ [53,54]. Another recent concept is the inducible SALT (iSALT), which denotes that the leukocytes (such as perivascular macrophages, dermal dendritic cells and T cells) involved in this structure are activated by local inflammatory stimuli [55,56]. iSALT formation is associated with activation of perivascular macrophages by IL-1a, a cytokine produced by keratinocytes in response to inflammatory agents. The activated macrophages produce CXCL2 (chemokine (C-X-C motif) ligand 2), a chemoattractant chemokine that recruits dermal dendritic cells and promotes effective antigen presentation and activation of T cells in the skin [54,55,56,57].

It is also important to emphasize the immunological relevance of the skin-associated microbiota [58,59]. These microbes cooperate with the skin and immune cells in order to maintain tissue homeostasis, for example, contributing to the effective development of innate and adaptive immune responses [60,61]. The microorganisms residing in the skin can interact through antagonistic or synergistic relationships [61]. For instance, the presence of microorganisms that metabolize host proteins and lipids results in the production of bioactive substances that act by inhibiting the proliferation of invading pathogens. They do this through the induction of immune mediators, such as IL-1 and IFN-γ, released from keratinocytes and resident T cells, respectively [58,62]. In addition, some studies show that several commensal species act to inhibit the proliferation of other pathogenic (or opportunists) bacteria, such as the relationship between *Corynebacterium* sp./*S. pneumoniae*, *S. epidermidis/S. aureus* and *S. epidermidis/Propionibacterium acnes* [61].

### 2.1. Immune Cells in the Epidermis

The epidermis mainly consists of keratinocytes that are closely linked to each other, forming a barrier and limiting access to the internal environment [63]. The keratinocytes play essential roles in the inflammatory response due to the secretion of cytokines (TNF-α, IL-1, IL-6) and chemokines (TARC/CCL17) [64,65], modulating the functions of T lymphocytes [66]. The Langerhans cells (LCs) are presented in the upper layer and combine features of macrophages (self-renewal, embryonic origin) and dendritic cells (antigen presentation, dendrites) [67,68]. The antigens in the lower layer of the human epidermis are captured by inflammatory dendritic epidermal cells (IDECs) [69].

The human epidermal resident T cells participate in immune surveillance and quickly respond to antigens from pathogens or damaged host cells. These cells participate in wound healing by expression of insulin-like growth factor 1 (IGF-1) [70,71]. Further, the sweat glands (epidermal appendages) are able to secrete antimicrobial peptides [36]. Macrophages and memory B cells can be also found in the epidermis, while neutrophils are recruited in response to tissue damage [41,42,72,73,74]. 

The macrophages assume different phenotypes that play distinct roles in skin physiology [75,76,77]. Differentiation to each phenotype is dependent on the cytokine involved in each situation [78]. The classical macrophages (also called M1), activated by INF-γ, are involved in phagocytosis and the release of inflammatory mediators such as cytokines (IL-1β, IL-6, IL-12 and TNF-α) and reactive species (nitric oxide and superoxide). M1 macrophages are crucial for the antimicrobial response and are characterized by the expression of MHC (major histocompatibility complex) class II receptor called HLA-DR (Human Leukocyte Antigen–DR isotype) [76,78,79,80].

The alternative activation of macrophages (M2) is trigged by IL-4, IL-5 and IL-13 (Th2 characteristic cytokines) and these macrophages express CD163 and arginase. This phenotype produces IL-10 and TGF-β, which are involved in the later phase of healing, promoting tissue repair [75,76,78,81]. In addition to their involvement in the reverse migration of neutrophils, the M2 macrophages induce fibroblast proliferation and collagen production [77,82,83]. Finally, the recently described M4 macrophages (induced by the chemokine CXCL4) are involved in the pathogenesis of skin lesions induced by *Mycobacterium leprae* [84].

Neutrophils are the first cells attracted to the wound site in response to chemotactic factors released by the skin resident cells (macrophages and keratinocytes), DAMPs and lipid mediators (such as leukotriene B4) [47,83,85]. These cells are responsible for the processes of sterilization and the degradation of cell debris through phagocytosis, neutrophil extracellular traps (NET) and the secretion of antimicrobial peptides and inflammatory mediators (reactive species and cytokines) [9,10,46,83].

Neutrophils also produce serine proteases and matrix metalloproteinases (MMPs); enzymes that are crucial for the correct progress of the healing process [86,87]. However, the high activity of these enzymes, along with the exacerbated release of inflammatory mediators, can promote tissue damage and contribute to the formation of chronic wounds [47,88,89]. Neutrophils are essential for effective wound healing by influencing M2 polarization through the release of cytokines and soluble factors (azurocidin, cathepsin G, colony stimulating factor 1, IL-13) [90,91]. The uptake of dead neutrophils and the secretion of microvesicles can also trigger the release of anti-inflammatory cytokines (such as TGF-β) by macrophages and promote wound repair [82,91]. 

Regarding B cells, recent data indicate that skin subpopulations are different from lymph node B cells, and that they are important in the regulation of inflammation and wound healing [73,92]. Immunosuppressive functions are attributed to a subset called B regulatory cells (Bregs) that produce anti-inflammatory cytokines IL-10, IL-35 and transforming growth factor-β (TGF-β), thus limiting the activation of inflammatory cells [93,94,95]. Bregs are involved in the differentiation of regulatory T cells [95].

### 2.2. Immune Cells in the Dermis

The dermis is composed of extracellular matrix proteins that give structure and elasticity to the skin. This structure provides nutrients and circulatory support to the epidermis [41,53]. Fibroblasts are the main cell types of the dermis that perform the synthesis of collagen, elastin and amorphous—fundamental substances for extracellular matrix formation [41]. These cells repair injured skin by providing structural support and guiding the migration of immune cells, allowing important cell–cell contact [96,97]. 

Fibroblasts are also able to produce cytokines (such as IL-1β, IL-6) and chemokines (such as CXCL8 and CXCL11) [98,99]. These mediators can actively recruit leukocyte subpopulations of the innate immune system, such as plasmacytoid dendritic cells (pDC), neutrophils, mast cells, and macrophages. This step is a crucial for the inflammatory events that follow the course of chronic inflammation, such as in the atopic dermatitis [100,101,102,103].

Other cells present in the dermis include natural killer cells, B lymphocytes and T lymphocytes [104]. T regulatory cells (Treg) are attracted by chemokine CCL20, which is produced in response to commensal microorganisms present in the skin after the birth [39]. Treg cells interact with Langerhans cells and are involved in the resolution of skin inflammation, promoting the proper healing process [105,106]. Langerhans cells also inhibit Treg during microbial invasion in order to promote inflammatory defense, which also includes the proliferation of memory cells [106].

## 3. ESKAPE and Wound Infections

As mentioned earlier, pathogenic microorganisms significantly slow the healing process due to tissue destruction that leads to an exacerbated immune response condition, characterizing chronic wounds [48,50]. In the following sections, some virulence determinants directly related to the action of ESKAPE pathogens in skin infections are discussed. We also address the antibiotic resistance genes that are more prevalent in the isolates related to skin infections. 

### 3.1. Enterococcus faecium and Related Species 

*Enterococcus* (Enterococcaceae family) species are predominantly non-pathogenic gastrointestinal commensal bacteria that, in certain circumstances, cause infections. However, some species of the genus have shown clinical relevance in the last decade, such as *Enterococcus faecalis* and *Enterococcus faecium*, both involved in wound infections [107,108,109]. Additionally, *Enterococcus gallinarum* and *Enterococcus casseliflavus/flavescens* (with intrinsic resistant towards vancomycin) have also gained attention due their involvement in surgical wound infections [110,111,112,113]. 

*E. faecium* is the representative member of *Enterococcus* genus in the ESKAPE group [114]. It has been isolated in surgical site infections and diabetic foot [115,116]. For example, a study conducted in India showed that *E. faecium* was the most commonly observed *Enterococcus* species in traumatic skin wounds; followed by *E. faecalis* [111]. Similarly, another study reported that *E. faecium* was the main etiologic agent in skin and soft-tissue infections (SSTI) related to combat casualties [117]. *E. faecium* is also involved in polymicrobial infections with *E. coli* [116] and *E. faecalis* [111]. Specifically, the association of *E. faecium* in diabetic foot ulcers was shown to be related with limb loss [116].

In addition, a recent study showed that some diabetic patients with wounds infected by *Enterococcus* presented evolution to osteomyelitis. This was a retrospective study of 275 patients with diabetic foot admitted at a tertiary care hospital in the UK in 2015. *Enterococcus* species, including vancomycin-resistant enterococci (VRE) strains accounted for 17% of Gram-positive isolates [118].

#### 3.1.1. Main Genes Involved in *Enterococcus faecium* Resistance 

*E. faecium* covers highly virulent strains, such as those of the clonal complex 17 (CC17), which have a multiple antimicrobial resistance profile due to the presence of several genes, such as *vanA* (vancomycin resistance) and *poxtA* [119,120,121]. This latter gene comes from mobile elements and has been frequently reported to confer resistance to phenicols, tetracycline and even linezolid, the last drug of choice for VRE strains [122,123,124]. *E. faecium* has the ability to survive in highly hostile and nutrient-poor environments [125,126]. These characteristics are also observed for *E. faecalis* strains [127].

Both *E. faecium* and *E. faecalis* have intrinsic resistance to cephalosporins, aminoglycosides, clindamycin, and trimethoprim/sulfamethoxazole [128,129,130]. VRE strains are recognized as major issues and it is estimated that these strains were responsible for infections in over 16,000 people with over a thousand deaths (1081) in European countries in 2015 [131]. Moreover, MDR enterococci are also present in coastal and fluvial waters, which can become a major public health problem due to the possibility of their transfer and arrival in the clinic [132].

Vancomycin resistance in *E. faecium* is related to *van* gene clusters that comprise the operons *vanA*, *vanB*, *vanC*, *vanD*, *vanE*, *vanG*, *vanL*, *vanM* and *vanN* [133,134,135]. Some *vanA* and *vanB* accessory genes (*vanY*, *vanZ* and *vanW*) have also been described and the entire cassettes can be carried by transposons (Tn*1546*, Tn*1547* or Tn*1549*) [136,137]. All these genes are easily deposited on plasmids or inserted directly into the major chromosome [138,139]. The *van* operons are also involved in resistance to other drugs, as they have genetic variability that enables a variety of resistance phenotypes [133].

Further, specific resistance to aminoglycosides, especially gentamicin, is provided by aminoglycoside modifying enzymes (AMEs) which are encoded by genes such as *aac(6’)-Ie-aph(2”)-Ia*, *aph(3’)-IIIa* and *ant(4’)-Ia*, which make it impossible to bind the drug to bacterial ribosomes and consequently, the protein synthesis is inhibited [140]. AMEs are also involved in resistance towards erythromycin, tetracycline and ciprofloxacin [141]. 

Recently, the prevalence of *E. faecalis* was determined in a study involving 200 surgical wound samples obtained from patients of Minia University hospital, Egypt. A frequency of 24 (12%) was reported for wound samples. All *E. faecalis* isolates were classified as MDR. Specifically, the rates of resistance towards erythromycin, vancomycin and linezolid were 100, 58.28 and 23.1% of the isolates from wound samples, respectively. In addition, the *vanA* gene was detected in 71.4% of vancomycin-resistant isolates. Similarly, the majority of the strains harbored resistance genes *ere(B)* and *erm(B)—*83.3% and 70.8%, respectively—responsible for the production of esterase enzymes for erythromycin [142].

The combination of existing drugs is an important strategy towards drug resistant strains of *E. faecium* and related species. The use of daptomycin with β-lactams has been well documented against VRE strains, including in murine models of infection [143,144,145]. Combinatory treatment with linezolid, quinupristin-dalfopristin, tigecycline and, more recently, oritavancin and dalbavancin also showed excellent results against resistant strains [146]. The combination of retapamulin with erythromycin, quinupristin/dalfopristin and quinupristine also demonstrated synergistic activity against *E. faecalis* [147]. Furthermore, the use of new drug candidates has also been explored. Compounds such as 1,2,4-oxadiazoles are considered to have therapeutic potential for the treatment of *E. faecium* MDR strains [148]. Similarly, 1,2,4-triazolo[1,5-a] pyrimidines were able to prevent the cell wall biosynthesis of *E. faecium* [149]

#### 3.1.2. Main Genes Involved in *Enterococcus faecium* Virulence

*E. faecium* also display a vast repertoire of virulence determinants that are involved in tissue adhesion and cytotoxicity. The production of biofilms by *E. faecium* is associated with multiple factors, such as *esp* (suggested as the major virulence determinant) which encodes the *Enterococcus* surface protein (Esp) which is related to adhesion to epithelial cells, as well as the secretion of aggregating substances [132,150]. Recent studies on the N-terminal region of Esp suggested that this protein acts by a mechanism involving amyloid-type aggregation to build the biofilm matrix in an acid environment [151]. 

Other virulence factors related to *Enterococci* adhesion and colonization include collagen-binding adhesin (encoded by *ace*), adhesin (*efaAfm*), cytolysin A (*cylA*), gelatinase (*gelE*), hyaluronidase (*hyl*) and emp pilus and aggregation substance (*asa1*) [132,152,153,154]. The collagen-binding proteins and cytolysins expressed by *E. faecium* compromise the bonds between collagen fibers and the balance between keratinocytes and fibroblasts [155]. Gelatinase and hyaluronidase are responsible for the hydrolysis of collagen fibers and the cutaneous extracellular matrix. The presence of an emp pilus, especially EmpA and EmpB subunits, is essential for the architecture of the pilus, formation and extension of biofilms, in addition to adhesion to fibrinogen and type I collagen [153]. Aggregating substances, on the other hand, facilitate the attachment to the skin epithelium and favor the bacterial aggregative behavior during plasmid conjugation [156]. 

A research study aiming to evaluate the genes associated with virulence and drug resistance determinants was performed with *Enterococcus* clinical isolates from burn patients. The authors reported a predominance of *E. faecalis* (80.7%) among the obtained enterococci (*n* = 57), while only two isolates were identified as *E. faecium*. The *E. faecium* strains were positive for *asa1*, *ace* and *gelE* [157]. Another study, also involving burn patients, reported a higher presence of *E. faecalis* (62.5%) and *E. faecium* (37.5%) among enterococcal isolates. These isolates had *gelE* and *asa* as the most detected virulence genes, while the *esp* and *cyl* showed a low level of detection. Only the *E. faecium* isolates exhibited resistance towards vancomycin and teicoplanin (24%). In general, higher levels of antibiotic resistance were observed in *E. faecium* [158]. 

The Table 1 illustrate some types of genes associated in resistance and virulence in *E. faecium.*

### 3.2. Staphylococcus aureus

*Staphylococcus aureus* naturally occurs in the microbiota of skin and other body tissues [159], facilitating the opportunistic infection of wounds [160,161]. In fact, *S. aureus* is one of the pathogens commonly isolated from skin lesions [162,163], with a high number of strains exhibiting complex combinations of virulence and resistance genes [3,164] (Table 2). It is an important causative agent of SSTIs, presenting high rates of morbidity and mortality, in addition to recurrent infections [165,166]. This species is also been related to the progression of diabetic foot to osteomyelitis [118].

The genomic variation in *S. aureus* is discontinuous, with distinct subdivisions called clonal complexes. The multifactorial forces that shape the variable structure in *S. aureus* are likely to include bacterial competition and barriers to genetic exchange [167,168]. Clones with high resistance to antibiotics and/or multiple virulence factors quickly emerge due to the acquisition of genes (by several routes) from other strains of *S. aureus* or even from other genera [169]. This plasticity allows *S. aureus* adaptation to different types of stress, enabling survival in different niches [170,171,172]. 

#### 3.2.1. Main Genes Involved in *Staphylococcus aureus* Resistance

An increasing number of *S. aureus* strains have been found to be resistant to antimicrobial agents. This genetic variability is mediated by a diverse set of mobile genetic elements (MGEs) that include plasmids, transposons, integrons, genomic islands, *S. aureus* pathogenicity islands (SaPIs), integrative conjugative elements, staphylococcal chromosome cassettes (SCC) and phages [173,174].

The first resistance episode by *S. aureus* was reported in the 1940s for penicillin (PRSA), a period close to its own discovery and use [175]. This type of resistance has been attributed to the *blaZ* gene, which encodes a specific type of β-lactamase, able to cleave penicillin through the hydrolysis of its β-lactam ring [176]. Thereafter, the rate of emergence of methicillin-resistant *S. aureus* (MRSA) and multidrug-resistant *S. aureus* (MDRSA) strains has been high in SSTI in hospitalized individuals [177,178]. Although traditionally linked to the hospital environment, some MRSA and MDRSA strains have emerged in the community and have caused severe cases of skin infections [179]. This phenomenon has increased the frequency and the severity of infection by this microorganism [180].

The high prevalence of MRSA in hospitals and community settings has been a major public health challenge worldwide. An American study reported that at least 72,000 cases of invasive MRSA infections were recorded in US health systems in 2014 [181]. The acquisition of methicillin resistance occurs through the presence of *mecA*, a gene encoding a penicillin-binding protein (PBP2a) [176].

The discovery of *mecA* was only possible twenty years after the appearance of the first cases of MRSA [182]. This gene is transported by mobile elements called Staphylococcal Chromosomes Cassette *mec* (SCC*mec*) [183]. At least eleven SCC*mec* types have been described and correlations between more virulent strains of MRSA and SSC*mec* types III and IV have been observed [184,185]. SCC*mec* can be carried by phages [186,187]. In addition, a French study that investigated the prevalence of fluoroquinolone-resistant staphylococci (FQR) in hospitalized and healthy patients showed that this type of resistance is also associated with MRSA strains [188].

Alarming levels of resistance are already detected in isolates of *S. aureus* for drugs considered as the last choice for treatment, such as vancomycin [189,190]. Originating from a conjugative plasmid, resistance to vancomycin is conferred by the VRE operon *vanA* (previously mentioned), where the entire original enterococcal plasmid is conjugated or only the transposon Tn*1546* is assigned to a resident plasmid of *S. aureus* [176].

The mechanism of action of vancomycin is based on the inhibition peptidoglycan polymerization, an important structural component of the bacterial cell wall [191,192]. The *vanA* operon—composed of the *vanA*, *vanH*, *vanX*, *vanS*, *vanR*, *vanY* and *vanZ* genes—is responsible for inhibiting the binding of vancomycin to peptidoglycan precursors, by either not synthesizing them or hydrolyzing those that already exist. This is regulated by a two-component system encoded by the *vanS* and *vanR* genes that activate the transcription of the operon [176].

Some other frequently reported phenotypes include borderline oxacillin-resistant *S. aureus* (BORSA) and vancomycin-resistant *S. aureus* (VRSA) strains. BORSA isolates are susceptible to cefoxitin and do not carry the *mecA* gene, but they are able to produce excessive amounts of β-lactamases, resulting in antimicrobial resistance [193,194]. It was also observed that resistance to oxacillin even alters the primary characteristics of the *S. aureus* biofilm and its virulence [195]. Strains resistant to vancomycin—one of the standard treatments for infections caused by MRSA—are emerging, and their behavior is attributed mainly to the *vanA* operon present in a plasmid derived from enterococci [176]. VRSA isolates have already been identified in skin lesions and diabetic foot ulcers [196]. 

Other MDRSA strains described harbored several resistance genes, such as: *blaR1*, *blaIe*, *lmrS*, *vraR*, *mrgA*, *qacA* and *qacB* for oxacillin and ciprofloxacin; *NorA*, which belongs to the major facilitator superfamily (MFS), and *MepA*, which belongs to the multidrug and toxic compound extrusion (MATE), for ciprofloxacin and norfloxacin; *MdeA* (MFS), related to resistance to novobiocin, mupirocin and fusidic acid; and *LmrS*, which encodes multiple drug efflux pumps, associated with trimethoprim and chloramphenicol [197,198,199,200].

As an alternative to the high rates of resistance and emergence of MDR strains, several new drugs have been used to treat infections caused by *S. aureus*. Recently, several agents have been approved to treat MRSA-infected skin lesions, including the lipoglycopeptides dalbavancin, oritavancin and telavancin, ceftaroline and tedizolid [201]. Other examples of new drugs include tannic acid, ivermectin and quinupristin/dalfopristin, which demonstrate success in combating *S. aureus* strains that are resistant to methicillin, erythromycin, ciprofloxacin, rifampicin and gentamicin [202,203,204].

Similarly, preliminary studies have shown that peptides such as nisin, AP7121, CSαβ-DLP2 and DLP4 demonstrate antibacterial effects against *S. aureus* (including MDRSA and VRSA strains). These compounds act by interrupting the molecular synthesis and microbial cell cycle [205,206,207]. Several natural products are also reported to have promising in vivo antimicrobial activity against *S. aureus*, including in models of wound infections [208,209,210,211].

#### 3.2.2. Main Genes Involved in *Staphylococcus aureus* Virulence

As previously mentioned, *S. aureus* can express a variety of virulence factors that facilitate cell adhesion, mediate evasion from the immune system and induce damage to host cells [161,173,212]. Adhesion to host cells is ensured by proteins that bind to fibronectin (FnbA and FnbB), collagen (Cna), fibrinogen (Fib), laminin (Eno) and elastin (EbpS). These proteins can be referred as microbial surface components recognizing adhesive matrix molecules (MSCRAMMs) that play important roles in the evasion of immune defenses and biofilm formation [213,214].

Indeed, great capacities for both biofilm formation and intracellular survival are described for *S. aureus* [165,166]. These properties are related to the firm and recalcitrant polysaccharide matrices that increase its virulence and resistance to antibiotics and may even contribute to bacterial survival in phagocytes (neutrophils and macrophages). Taken together, these factors contribute for the spreading of *S. aureus* and predispose an infected individual to chronic and persistent infection [215,216].

Other virulence factors extremely relevant to skin infections are the toxins secreted by *S. aureus* that provoke tissue damage and abscess formation [217]. Among them is α-toxin, a 33 kDa pore-forming cytolytic protein which affects a wide range of human cell types, including epithelial cells, endothelial cells, T cells, monocytes and macrophages [218]. In this sense, in addition to tissue damage, this toxin is able to neutralize the protective immune response [217].

Exfoliative toxins (ETs) and leukocidins—including leukocidin ED (LukED) and Panton-Valentine leukocidin (PVL)—also play important roles in the pathogenesis of *S. aureus* as they destroy cell membranes by creating β-barrel-like pores that lead to cell lysis. Additionally, they impair the activation of resident immune cells [219,220]. ETs selectively cleave peptide bonds in the extracellular region of human desmoglein-1, which acts as an adhesion molecule between keratinocytes. ETs are related to staphylococcal scalded skin syndrome and bullous impetigo [221,222]. The three ETs are encoded by different genetic regions: *eta* (found in a phage), *etb* (located on plasmids), *etd* (located on genomic islands) [221].

LukED (encoded by the *lukED gene*) is described as a major cause of blood and skin infections, such as impetigo [220]. In turn, the *pvl* gene is commonly detected in *S. aureus* strains isolated from SSTI and its product is responsible for the destruction of resident immune cells and tissue necrosis [219,221]. Several reports have shown a high prevalence of MRSA carrying the *pvl* gene in community-acquired SSTI, where some of the wounds required surgical procedures for incision or drainage, and many of these strains were also resistant to erythromycin, clindamycin and tetracycline [223,224,225].

The frequency of detection of the *pvl* gene may vary according to the region and clonal group of *S. aureus*—related to community-associated methicillin-resistant *Staphylococcus aureus* (CA-MRSA) strains in many countries. For instance, in a study carried out in Nigeria, a high frequency of *pvl* gene detection was observed for SSTI and wounds, with rates 83.3 and 79.2%, respectively [226]. However, another study carried out in Iran reported that the frequency of the *pvl* gene was 33.3% for CA-MRSA isolates obtained from infected wounds [227]. 

Phenol-soluble modulins (PSMs) have gained attention for their involvement in the inflammatory response, inducing the production of cytokines and neutrophil migration [228,229,230]. During skin infection, PSMα released by *S. aureus*, has also been shown to influence the levels of IL-17 produced by keratinocytes [229]. It is believed that serious SSTI associated with CA-MRSA strains, may be related to the cytotoxic and membrane-disturbing PSMα [231]. PSMα, secreted by CA-MRSA, can induce the rapid formation of a type of NET that is related to the destruction of phagocytic cells, rather than contributing to the death of pathogens [231]. 

A recent study evaluated the presence of virulence and resistance-related genes in isolates of *S. aureus* from samples of skin infections (*n =* 200). A total of thirty-six (18%) isolates with the MDR profile carried the *mupA* gene, the predominant determinants of virulence included PSMα (61.5%), *pvl* (2.5%), *eta* (2.5%) and *etb* (1%) [232].

In addition, *S. aureus* has mechanisms that are directly involved in processes related to immune response modulation, such as: SSL3 (staphylococcal superantigen-like protein 3), an inhibitor of neutrophils and other TLR2-expressing cells [233]; proteases with several targets (complement system, LL-37) [234,235]; staphylokinase protein (SAK), an inhibitor of LL-37, and α-defensins [236,237].

Some examples of genes involved in virulence and resistance of *S. aureus* are summarized in Table 2. 

### 3.3. Klebsiella pneumoniae

*K. pneumoniae* is an opportunistic, Gram-negative, encapsulated and cosmopolitan pathogen that usually causes skin infections in burned and/or immunocompromised individuals, often forming thick biofilms [238,239]. It is considered to be one of the main causes of health-associated infections [240]. For instance, studies conducted at the US Army Surgical Research Institute (Burn Center), showed *K. pneumoniae* as one of the four major pathogens isolated from infected wounds in hospitalized burn patients [241,242]. Similarly, an epidemiological analysis showed that 15.1% of hospital isolates of *K. pneumoniae* from Turkey came from cutaneous lesions [243].

#### 3.3.1. Main Genes Involved in *Klebsiella pneumoniae* Resistance

The emergence of *K. pneumoniae* strains with hypervirulent phenotypes (hvKp) and more aggressive capsular serotypes, such as K1 and K2 present in CC23 (clonal complex 23), has been observed to be more frequent in severe skin and soft tissue infections [244,245]. Even the reduced use of antibiotics such as aminoglycosides proved to be sufficient for the emergence of resistance phenotypes, such as the expression of the *armA* gene, which encodes the 16SrRNA methylase enzyme responsible for blocking binding to the bacterial ribosome [246]. Other aminoglycoside resistance genes that have already been reported include *aacA4*, *aacC2* and *aadA1* [247] (Table 3).

In addition, the appearance of carbapenem-resistant *K. pneumoniae* (CRKP) strains has made it very difficult to treat burn wounds that are infected with this pathogen [248]. CRKP strains express carbapenemases, a type of enzyme (encoded by genes including *bla*_KPC-2_ and *bla*_KPC-3_) that is able to cleave the β-lactam drugs and exhibits low susceptibility to the action of beta-lactamase inhibitors (clavulanic acid and tazobactam) [249,250,251].

*K. pneumoniae* strains also show resistance to third generation cephalosporins and fluoroquinolones, mediated by extended spectrum β-lactamases (ESBLs) [245,252]. Allied to this, changes in cell permeability represent the main mechanism involved in resistance to quinolones, through the expression of the *acrAB* gene—responsible for efflux pumps [246].

A Chinese survey evaluated the prevalence of carbapenem-resistant Enterobacteriaceae in various types of infection. The study showed that carbapenem-resistant *K. pneumoniae* was the most common etiologic agent of deep wound infections (85.7%); while it was detected in 18.8% cases of superficial wound infections. Considering all types of infections, a high prevalence of nosocomial carbapenem-resistant *K. pneumoniae* producing IMP-4 carbapenemase (84%) and IMP-8 carbapenemase (50%) was detected [253].

Recently, research was performed that phenotypically and molecularly characterized *K. pneumoniae* isolates that were obtained from wounds of hospitalized patients in Tehran, Iran. The authors reported that 45.1 and 22.5% were producers of extended-spectrum β-lactamases (ESBL) and carbapenemase, respectively [254]. The isolates simultaneously carried the genes encoding ESBL (78.4%), AMEs (*aac(6’)-Ib*; 65.7%), carbapenemase (50%) and quinolone resistance determinants (QDRs; 49%). The authors of this study highlighted that four isolates carried the genes for carbapenemases (bla_TEM_, bla_SHV_, bla_CTX-M_), QDRs (*qnrB* and *qnrS*) and *aac(6’)-Ib* [254].

Some strains of *K. pneumoniae* have even shown resistance to last generation antibiotics, such as polymyxin. Reductions in negative ions hinder the binding of the drug to the bacterial surface. This occurs due a chromosomal system of modifications in the lipopolysaccharides (LPS). These changes are attributed to central genes involved in lipid A synthesis, such as *lpxM* [246]. A study carried out in Korean hospitals also showed that 16% of the samples of *K. pneumoniae* collected were resistant to tigecycline, a drug used to treat MDR strains, with mutations in the *ramR* and *rpsJ* genes and massive expression of the *tetA* gene also being documented [255].

In view of this great resistance problem and the appearance of MDR strains, the treatment of *K. pneumoniae* becomes quite challenging. An ideal therapeutic protocol for infections caused by Multidrug-resistant *K. pneumoniae* (MDR-KP) has not yet been well defined, but the use of high-dose meropenem, fosfomycin, tigecycline, aminoglycosides and polymyxins is widespread [256]. The combination of colistin with niclosamide has even shown good results against strains that are already resistant to colistin itself [257].

In addition, several drugs are in the clinical stages of testing for MDR-KP, with good results having been produced so far. The association of these new candidates with marketed drugs is seen as having great potential in the fight against these pathogens. These include: ceftazidime-avibactam, meropenem-vaborbactam, imipenem-relebactam, plazomicin, cefiderocol, aztreonam-avibactam, ceftaroline-avibactam, cefepime-zidebactam and nacubactam [256]. Further, immunotherapies have been evaluated, such as the investigation of cystatins 9 and C as a solution for *K. pneumoniae* strains producer of metallo-β-lactamase-1 [258]. 

#### 3.3.2. Main Genes Involved in *Klebsiella pneumoniae* Virulence

Currently, four factors have been well described as contributing to *K. pneumoniae* virulence: fimbriae (important for the formation and installation of biofilms), capsule, lipopolysaccharide and iron uptake [240]. For the effective formation of a biofilm, it is necessary that the microorganisms involved become close enough to the target surface, fixing themselves to it with the aid of fimbriae and/or a flagella [259]. In *K. pneumoniae*, fimbriae type 1 and 3 are encoded by the operon *mrkABCDF*, and the subunits MrkA and MrkD are directly involved in binding to collagen [260,261].

The polysaccharide capsule (encoded by the capsular polysaccharide synthesis locus—*cps* gene) is another important virulence factor for the establishment of skin infections, since it prevents phagocytosis and complement-mediated killing [262,263]. For *K. pneumoniae*, 78 capsular serotypes have been reported. In particular, K1 and K2 confer hypervirulence through the excessive production of hypermucoviscous capsular material [240]. The *rmpA* gene—located in plasmids of *K. pneumoniae*—has also been observed to be an important virulence factor and is responsible for the synthesis of capsular compounds [246].

Candan et al. (2015) also described the importance of various genes for the production of the *K. pneumoniae* capsule (*magA*, *k2A* and *wcaG*) and its lipopolysaccharides (*wabG*, *uge* and *ycfM*), such as LPS. The products of these genes are essential for the formation of the thick and consistent biofilms that are frequently found in the skin and soft tissue infections of burned or immunocompromised patients [264].

The outermost parts of the LPS, named O-antigens, are also used for the serotyping of *K. pneumoniae*. At least, nine O-antigen serotypes have been described and these structures are considered as representing potential targets for vaccination [265,266]. A study using a global collection of *K. pneumoniae* showed that the serotypes O1, O2 and O3 were most prevalent in all types of infections [267]. The O-antigen in the O1 serotype is composed of D-galactan I and D-galactan II, with the latter being recognized as the immunodominant antigen. The synthesis of D-galactan II is performed by glycosyltransferases WbbY and WbbZ. Interesting, D-Gal II is more prevalent in community-acquired pyogenic liver abscess (PLA) strains than in non-tissue-invasive strains [268]. In addition, some strains of *K. pneumoniae* can modify the composition of LPS, avoiding recognition by the TLR4 receptors [240]. 

As a crucial factor for the growth and infection process of *K. pneumoniae*, the production of enterobactin, mediated mainly by the *entS* gene, is directly related to the ability to chelate iron molecules from the host (siderophores). The presence of enterobactin is observed in normal and hypervirulent strains, while other molecules also involved in the iron absorption process, such as aerobactin, yersiniabactin and salmoquelin are more common only in hypervirulent strains [240,269].

Some examples of virulence and drug resistance-related genes reported for *K. pneumoniae* are provided in Table 3.

### 3.4. Acinetobacter baumannii

*A. baumannii* is an important opportunistic nosocomial pathogen that causes severe infections associated with ventilation and blood flow in critically ill patients, but also serious infections in patients with skin lesions, especially burns [270,271,272]. MDR *A. baumannii* (MDR-AB) has been associated with severe and fatal cases of SSTI [273,274,275].

A recent study highlighted that 15% of patients admitted to a particular hospital acquired nosocomial infections due to MDR-AB, a condition associated with prolonged hospitalization and increased risk of death [275]. In addition, a one-day cross-section showed the presence of *A. baumannii* DNA in 10% of the individuals tested [276].

*A. baumannii* is often found in skin and soft tissue infections resulting from burns, mechanical trauma or the wounds of soldiers from war situations. There have been reports of osteomyelitis and exposed tibial fractures caused by MDR-AB during military combat operations [277,278]. Another study that evaluated a burn care center registered that the presence of *A. baumannii* correlated with the worsening of health status of the infected patients. In this case, higher levels of morbidity and mortality and longer hospital stays being documented in comparison to patients with uncontaminated wounds [279].

#### 3.4.1. Main Genes Involved in *Acinetobacter baumannii* Resistance

The resistance of *A. baumannii* to β-lactam antibiotics is mainly mediated by enzymatic hydrolysis [18]. High cleavage capacities have been observed in all known penicillins and cephalosporins (except cephamycin) by the following β-lactamases: CTX-M (encoded by bla*_ctx-m_*gene), GES (*bla_ges_*), PER (*bla_per_*), SCO (*bla_sco_*), SHV (*bla_shv_*), TEM (*bla_tem_*) and VEB (*bla_veb_*) [280,281,282,283] (Table 4). It was also observed that *A. baumannii* has an extreme tolerance to free radicals (such as hydrogen peroxide), as it has ample genomic flexibility and contains the element *ISAba1* upstream of the catalase *katG* gene, which is responsible for improving resistance [284].

On the other hand, as in most Gram-negative bacteria, the emergence of resistance to tetracycline in *A. baumannii* occurs through efflux pumps [285]. In this case, these are Tet type pumps, encoded by the *tetA* gene; whereas those encoded by the *tetB* gene confer resistance to tetracyclines and also minocycline and doxycycline. Resistant strains of *A. baumannii* carrying the ribosomal defense gene *tetM* have already been identified [18]. For fluoroquinolones-resistant *A. baumannii* strains, mutations have been observed in specific drug targets, especially in the *gyrA* of DNA gyrase and *parC* of topoisomerase IV genes [286]. Episodes of resistance by light chromosomal efflux pumps have also been reported [287].

Regarding resistance to aminoglycosides, different types of AMEs are synthetized by *A. baumannii* strains such as phosphotransferases, acetyltransferases and adenyltransferases. These enzymes are encoded by genes such as *aac(3′)-Ia*, *ant(2’)-Ia* and *ant(3″)* In addition, *A. baumannii* strains can harbor genes for several types of AMEs at the same time [288,289]. Resistance to aminoglycosides can also occur through the expression of the *armA*, *rmtA*, *rmtB*, *rmtC* and *rmtD* genes, that alter the binding to bacterial ribosomes [18]. Specifically, two efflux pumps can also affect the action of gentamicin: AdeABC and AbeM [290].

Alterations in the *pmrCAB* operon of *A. baumannii* are directly related to resistance to polymyxins, such as colistin. The *pmrC* gene encodes a modifying phosphoethanolamine transferase, while *pmrA* and *prmB* regulate a two-system regulatory mechanism. Mutations in these last two systems favor the expression of *pmrC* that is responsible for modifying lipid A [291]. More recently, it has also been reported that the *lpsB*, *lptD* and *vacJ* genes reduce fluidity and increase the osmotic resistance of the outer membrane of *A. baumannii*, inducing resistance to polymyxin A [292].

Confirming these facts, a cross-sectional descriptive study carried out in five hospitals in Medellin over a period of 2 years detected 32 patients with infections caused by MDR strains of *A. baumannii*. The incidence of SSTI and osteomyelitis was 21.9 and 18.7%, respectively. The authors reported a high rate of antibiotic resistance among most isolates (80%), with genes for carbapenemases *oxa_-23_* and *oxa_-51_* detected in all strains of skin lesions [293]. A Brazilian analysis on osteomyelitis also showed that *A. baumannii* was present in 21% of cases, with 40% of these being resistant to carbapenems [294].

Phage-based therapies have displayed promising results against MDR-AB strains. In vivo models showed that phage-based therapy was successful in containing infections caused by multidrug-resistant *A. baumannii* [295,296,297]. Ultraviolet C light, blue light, and pimenta oil have also showed interesting activities in mice models of wound infections caused by *A. baumannii* [298,299,300].

Moreover, the combination of existing drugs is widely adopted for the treatment of injuries caused by MDR-AB. For instance, combined therapy using colistin and niclosamide has proven to be effective against strains that are already resistant to colistin [257]. Similarly, nisin was also shown to potentiate the action of polymyxin B against resistant *A. baumannii* [301]. Another alternative is the association of protegrin-1 with colistin, fosfomycin, levofloxacin, meropenem, tigecycline and rifampicin in the treatment of surgical wounds colonized by *A. baumannii* [302].

#### 3.4.2. Main Genes Involved in *Acinetobacter baumannii* Virulence

Currently, the literature converges in affirming that *A. baumannii* has about 16 gene islands associated with virulence factors, thus directing a good part of its genome to pathogenic processes [303]. The main virulence factors associated with *A. baumannii* include systems of protein secretion, phospholipases, LPS, elements attached to the outer membrane, quorum sensing for biofilms and metal absorption [304].

Protein secretion systems are very effective in the virulence of *A. baumannii*. OmpA (encoded by the *ompA* gene) is one of the most studied proteins, as it is involved in the adhesion of epithelial cells and plays essential roles in the regulation of aggregation and biofilm formation in SSTIs [305,306]. Therefore, this protein represents a target for new antivirulence approaches against this pathogen [306].

OmpA is directly related to the mechanisms of cellular invasion and apoptosis, with an essential function in penetration of small solutes, and being classified as a single integral membrane protein anchored in the outer membrane [306,307]. The fixation and formation of biofilms by *A. baumannii* can occur in two ways: reversibly, where there is a strong physical–chemical attraction force, fundamental for the interaction between the strains and the contact surface; and irreversibly, as a result of the production of a matrix rich in exopolysaccharides, that is responsible for the permanent and coordinated adhesion of pathogens [308,309].

Phospholipases are other virulence factors widely described in *A. baumannii*. They are characterized as very important lipolytic enzymes for the cleavage of phospholipids that are present in cell membranes [310]. An example is phospholipase C that contributes to the cytolytic activity, allowing entry into epithelial cells [311,312]. Compounds coupled to the outer membrane of *A. baumannii*, such as the LPS antigenic O-polysaccharide, Csu pili (encoded by the *csu* gene) and biofilm-associated proteins (BapAb, encoded by the *bap* gene) can further promote adherence to skin epithelial cells as an initial stage of the colonization process [18,313].

*A. baumannii* is also known for having an external capsule with a high water-holding capacity, characterized by a dense polysaccharide that covers the entire surface of the bacterial cell and protects it against hostile environments, for example, dryness, disinfection and phagocytosis [314,315,316]. In addition, the synthesis of acinetobactin in a murine model of infection has been described, with an aggressive virulence factor of *A. baumannii* being noted in SSTIs [317]. *A. baumannii* also has sophisticated systems for metal acquisition. For example, in response to zinc (Zn), the pathogen can activate the expression of Zig A (a Zn-binding GTPase) encoded by *zigA* gene. The Zn uptake sytem is also composed by the ABC transporter and TonB, proteins presented in the inner and outer membranes, respectively) [318,319].

The genes associated in resistance and virulence in *A. baumannii* are represented in Table 4.

### 3.5. Pseudomonas aeruginosa

Undoubtedly, *P. aeruginosa* is one of the main pathogenic bacteria present in skin wounds. This microorganism belongs to the family Pseudomonadaceae, a Gram-negative bacterium that has the ability to develop in most natural and artificial environments [320]. This opportunistic pathogen is often isolated from samples of soil, water, plants and animals and can easily become resistant to antibiotics [321].

*P. aeruginosa* causes localized and systemic infections (e.g., ventilator-associated pneumonia, urinary tract infections or wound infections), especially in patients with severe burns, bet ulcers, and seriously ill and immunosuppressed subjects. Estimates indicate that this pathogen is involved in 10–15% of nosocomial infections, with a high prevalence of pulmonary complications in patients with cystic fibrosis [322,323]. *P. aeruginosa* is estimated to be present in at least one third of all skin infections worldwide, colonizing traumatic wounds, pressure and chronic ulcers and acantholytic or exudative dermatoses [324].

#### 3.5.1. Main Genes Involved in *Pseudomonas aeruginosa* Resistance

*P. aeruginosa* has several resistance mechanisms, which can be classified as intrinsic (e.g., decreased permeability, expression of efflux systems and changes in the target; acquired, through gene transfer and mutations) and adaptive (transient in the presence or absence of stressors) [322,325,326].

In intrinsic and acquired forms, *P. aeruginosa* limits the entry of antibiotics into its cytoplasm by reducing the amount of non-specific porins in the membrane and replacing them with more specific ones for essential nutrients; for example, the mutation of the porin OprD (*OprD* gene), that reduces permeability to carbapenems [325] (Table 5). Even when some harmful substances can penetrate the bacterial cell, *P. aeruginosa* is able to activate its highly complex multi-drug efflux pump systems. The four best described are: MexAB-OprM, encoded by the *mexAB-oprM* genes; MexXY/OprM (OprA), by the expression of the *mexXY-(oprA)* genes; MexCD-OprJ, by the *mexCD-oprJ* genes; and MexEF-OprN, by *mexEF-oprN* [326].

These mutations are so frequent that in Europe, the report of European Centre for Disease Prevention and Control (ECDC), published in 2016, showed that 33.9% of *P. aeruginosa* isolates were resistant to at least one of the currently used antimicrobial groups [322]. Resistance to the most commonly used classes of drugs—such as fluoroquinolones—by strains of *P. aeruginosa* can also be observed through mutations of the targets of these antibiotics, more specifically, mutations in the *gyrA* and *gyrB* genes of DNA gyrase and the *parC* and *parE* genes of topoisomerase IV [327].

The resistance of *P. aeruginosa* to potent polymyxins has also been reported, occurring via chromosomal mutations [328,329]. However, recently acquired resistance in these strains was also detected by means of plasmids, through the conjugation of the genes *mcr-1* and *bla_NDM-1_*, from *E. coli* and *K. pneumoniae*, respectively, both conferring resistance to colistin [330,331].

The adaptive mechanisms of resistance of *P. aeruginosa* have not yet been clarified. It is only known that this system depends on changes in defense gene expression in the presence of aggressive agents, and its withdrawal after a reduction in stress levels [332,333]. A shared characteristic for *P. aeruginosa* adaptive mutants is that they exhibit high levels of AmpC, due to the inactivation of *ampD* (*ampC* repressor) and other isolated *ampR* mutations, which assist in the coding of essential regulatory proteins in the induction of the *ampC* gene [326].

Thus, patients with burn infections caused by multidrug-resistant strains of *P. aeruginosa*, are generally affected by sepsis and suffer from high morbidity and mortality [334,335]. In a recent study, where 93 samples of *P. aeruginosa* collected from burn wound infections were isolated, 100% were resistant to one or more antimicrobials and 94.6% were multidrug-resistant [323].

Another major public health problem, resulting from infections by multidrug-resistant strains of *P. aeruginosa*, relates to the complications associated with diabetic patients [336,337]. In these patients, *P. aeruginosa* MDR has become an issue in the treatment of infections in diabetic foot ulcers (DFU) [338]. High rates of MBL-producing *P. aeruginosa* have been observed in many patients hospitalized with DFU, with this leading to lower limb amputation [337,338]. It has been described that the presence of the *exoS* and *exoU* genes is closely and directly related to the phenomena of antimicrobial resistance to multiple drugs and increased hospital stay length, making the individual more susceptible to pressure ulcers [339].

Some drug formulations have exhibited promising results towards multidrug-resistant *P. aeruginosa* strains in clinical trials. The associations between ceftazidime/avibactam and ceftolozane/tazobactam have shown excellent responses, including in phase III clinical studies [340,341,342,343,344]. The synergistic actions of these drugs with other drugs that are already used, such as meropenem, amikacin, aztreonam, colistin and fosfomycin, also demonstrated good results [345].

Additionally, the development of cefiderocol, a new siderophore Cephalosporin, represents a great hope for the treatment of injuries caused by MDR-PA [346,347]. The use of relebactam, imipenem and cilastatin, and some antibacterial peptides (such as ZY4) have also been demonstrated as alternatives in the fight against *P. aeruginosa* MDR [348,349]. Plant-derived compounds and probiotics have been suggested as emergent candidates for the treatment of *P. aeruginosa-*wound infections [350,351,352,353]. Finally, some studies have revealed the efficacy of some experimental vaccines for the prevention of skin infections by *P. aeruginosa* [354,355].

#### 3.5.2. Main Genes Involved in *Pseudomonas aeruginosa* Virulence

Collectively, the virulence factors of *P. aeruginosa* ensure the process of invasion, tissue colonization and damage, and dissemination in the bloodstream [323]. The virulence factors associated with bacterial cells include the flagella, lipopolysaccharide, pili type III system—effector proteins that include ExoS (*exoS)*, ExoT (*exoT*), ExoY (*exoY*) and ExoU (*exoU*)—and alginate. The extracellular determinants include hydrogen cyanide, metalloprotease zinc (LasB), alkaline protease, elastase (LASA), phospholipases (PLCH and PlcN), exotoxins and pyocyanin [323,339]. For the establishment of chronic infections, *P. aeruginosa* assumes a more aggressive behavior due adaptive mechanisms that involve the loss of fimbriae and flagella to isolate the host immune system and form biofilms. This state is associated with persistent inflammation, derived from the secretion of extracellular virulence factors [356].

*P. aeruginosa* possesses two different types of control systems that control the expression of the majority the virulence factors: the transcription regulatory system and two-component detection system-quorum. The two-component system (TCS) detects external signals by means of phosphotransferase, which activates specific transcriptional regulators, allowing cells to modulate gene expression in response to environmental conditions [320]. The expression of several virulence factors (including lipases, elastases, the skin and the production of many protease cytotoxins) is controlled by the mechanism of *quorum sensing*. This system has self-regulation dependent on the cell density. This mechanism favors the formation of aggressive and difficult to remove biofilms [320].

For example, it is known that elastase and alkaline protease (*phzI*, *phzII*, *phzH*, *phzM*, *phzS*, *plcH*a and *plcN* genes) deteriorate various components of the tissue—such as protein elements of connective tissue—and cleave the cell surface receptors of leukocytes, hindering the healing process of the skin [357]. It has also been observed that *P. aeruginosa* inhibits the degranulation of eosinophils that are present in the injured region, which ends up being an important inhibitory factor for the immune system, favoring constant tissue infection [358].

The expression of pili (*pilA* and *pilB* genes) participates in bacterial adhesion and the colonization of epithelial surfaces, as does the expression of the flagellum [357]. This induces an inflammatory response resulting in the production of IL-8, IL-6 and mucin [356,357]. Alginate plays a role in mediating mucin adhesion and promoting resistance to the defense mechanisms of the immune system, by inhibiting antibody binding and phagocytosis. The type III secretion system (TTS) injects various toxins directly into the cytosol of the host cells—with ExoU and ExoT known as being the most virulent [339,359]. Other extracellular virulence factors include phospholipase C, which destroys the host cell membrane, and exotoxin A (*oxA* gene), which contributes to both tissue damage in the early stages of infection, and to the uptake of important nutrients for its growth [357].

The Table 5 provides the examples of genes related for virulence and drug resistance in *Pseudomonas aeruginosa*.

### 3.6. Enterobacter spp.

Species from the genus *Enterobacter* are often associated with opportunistic skin infections in immunocompromised patients and demonstrate widespread resistance to antibiotics [22,360]. The most pathogenic species are usually referred to as *Enterobacter cloacae* complex (ECC), with the most commonly associated species being *E. cloacae* and *E. hormaechei,* in addition to *E. aerogenes*. *Enterobacter* is among the five most common Enterobacteriaceae involved in wound infections and SSTIs [361,362,363,364]. Some studies also point out the emergence of EEC clones with high epidemic potential [361,365,366]. Infection with *E. cloacae* or *E. aerogenes* results in mortality rates of up to 40% [361,367].

Despite its high prevalence, little is known about the virulence mechanisms of this genus of Enterobacteriaceae in SSTIs, but many mechanisms of antimicrobial resistance acquired by these microorganisms have already been reported [364] (Table 6).

#### Main Genes Involved in *Enterobacter* Resistance

Genetic analysis proved that ECC are producers of ESBL [220]. Several ECC strains have an MDR profile due the presence of enzymes that prevent the action of systemic and topical antibiotics that are used in the treatment of infected skin lesions, for example, TEM-1 β-lactamase [22,368,369]. The *bla*_TEM-1_ gene and its variants have high mutation rates which results in diversification of the enzymatic subtypes of resistance [22]. 

Alonzo et al. (2012) showed that 41.5% of the samples obtained by *Enterobacter* spp. were positive for the bla_CTX-M_ resistance gene, which showed greater activity against the cephalosporins cefotaxime and ceftazidime [220]. Another study showed *Enterobacter* spp. as the third most common microorganism found in the evaluation of patients with mild to extreme severe burn injuries with signs of infection in the skin [370].

Other *Enterobacter* species have also been considered as being highly pathogenic. For instance, an MDR strain of *E. asburiae* was detected that expressed resistance genes to aminoglycosides, β-lactams, fluoroquinolones, fosfomycin, macrolides, phenicols, rifampicin and sulfonamides. The gene *bla_IMP-8_* was located in the IncFIB plasmid, while *bla_CTX-M-3_* and *qnrS1* were both in the IncP1 plasmid. A non-typeable plasmid harbored *bla_CTX-M-14_*, *bla_TEM-1B_*, *bla_OXA-1_*, *catB3* (phenicols resistance) and *sul1* (sulfonamide resistance) [371]. Subsequently, *E. cancerogenus* was reported as a seriously aggressive infectious agent in skin wounds caused by mechanical trauma [372].

A larger survey involving 110 patients with skin ulcers infected by different microorganisms, showed that *E. cloacae* was present in approximately 7% of cases [373]. More specifically, all ten *E. cloacae* isolates obtained from a Turkish hospital were resistant to all available carbapenems; nine showed resistance to cefoperazone/sulbactam, trimethoprim and sulfamethoxazole, and 50–70% were resistant to other classes, such as aminoglycosides (gentamicin and amikacin) and fluoroquinolones (ciprofloxacin). The main resistance genes found in these samples were *bla_NDM_* (an unprecedented finding for this species), *bla*_VIM_ and *bla*_IMP_ [374]. Some strains of *Enterobacter* spp. also expressed *bla_KPC-2_*, *bla_KPC-3_*, *bla_KPC-4_* and *bla_NDM-1_* carbapenemic resistance genes [375].

Even with the high rate of emergence of resistant species in the *Enterobacter* genus, the clinical use of aztreonam has still shown good results in cases of severe infection by MDR clones, with no episodes of resistance reported to date [29]. The combination of colistin and imipenem drugs has also shown excellent results in in vivo models of infection [376]. Satisfactory results have also been achieved with the application of phage-based therapy (such as pyophages and multiple cocktails) in experimental models of infections induced by *Enterobacter* MDR strains [377,378]. The genes discussed in this section are summarized in Table 6.

## 4. Conclusions

This work discusses the main immunological resources involved in the skin’s defense against pathogens and highlights the importance of ESKAPE bacteria as etiologic agents of cutaneous infections. The high incidence of antimicrobial resistance and hypervirulent profiles observed for ESKAPE pathogens are associated with the difficulties in the treatment of the infections provoked by them. In addition, these bacteria are prevalent in hospital settings where they can affect immunocompromised patients. Knowledge about the virulence and resistance markers of these species is important in order to develop new strategies to detect and treat their associated infections.

## Figures and Tables

**Table 1:** Examples of virulence and drug resistance-related genes reported for the *Enterococcus faecium*.

Genes	Product Function	Reference
*Esp*	Product is *Enterococcus* surface protein (Esp) which is responsible for epithelial cell adhesion and increased binding between the polysaccharide matrix and collagen binding proteins.	[132,150]
*ace;* * efaAfm; cylA *	Encode collagen binding adhesin and cytolysins that compromise the bonds between collagen fibers and the balance between keratinocytes and fibroblasts.	[155]
*gelE*; *hyl*	Responsible for the hydrolysis of collagen fibers and the cutaneous extracellular matrix.	[132,152,153,154]
*Asa*	Encodes aggregating substances, which facilitate the attachment to the skin epithelium and favor the bacterial aggregative behavior during plasmid conjugation.	[156]
*vanA*; *vanB*; *vanC*; *vanD*; *vanE*; *vanG*; *vanL*; *vanM*; *vanN*	Vancomycin resistance.	[133,134,135]
*poxtA*	Phenicols, tetracycline and linezolid resistance.	[122,123,124]
*aac(6’)-Ie*; *aph(2’’)*; *aph(3’)-IIIa*; *ant(4’)-Ia*	Encode aminoglycoside modifying enzymes (AMEs) that confer resistance to drugs.	[140]
*ere(B)*; *erm(B)*	Responsible for the production of esterase enzymes for erythromycin.	[142]

**Table 2:** Examples of virulence and drug resistance-related genes reported for *Staphylococcus aureus*.

Genes	Product Function	Reference
*etA; etB; etD*	Encode the exfoliative toxins A, B and D that selectively bind and cleave a desmoglein-1 peptide bond.	[221]
*lukED*	Encodes leukocidin ED (LukED), a toxin related to blood and skin infections.	[220]
*pvl*	Encodes the Panton-Valentine leukocidin (PVL) which is associated to the destruction of resident immune cells and tissue necrosis.	[219,221]
*blaZ*	Involved in penicillin resistance, through the hydrolysis of its β-lactam ring.	[176]
*mecA*	Its product confers methicillin resistance, through a penicillin-binding protein.	[176]
*vanA*; *vanH*; *vanX*; *vanS*; *vanR*; *vanY*; *vanZ*; *blaR1*; *blaIe*; *lmrS*; *vraR*; *mrgA*; *qacA*; *qacB; norA*; *mepA*; *mdeA*; *lmrS*; *mupA*	These genes are involved in Multi-drug resistance—vancomycin, oxacillin, ciprofloxacin, norfloxacin, novobiocin, mupirocin, fusidic acid, trimethoprim and chloramphenicol.	[197,198,199,200]

**Table 3:** Examples of virulence and drug resistance-related genes reported for *Klebsiella pneumoniae*.

Genes	Product Function	Reference
*mrkABCDF*	Encodes fimbriae type 1 and 3; binding to collagen.	[260,261]
*Cps*	Encodes polysaccharide capsule.	[262,263]
*rmpA*	Synthesis of capsular compounds.	[246]
*magA*, *k2A*; *wcaG*; *wabG*; *uge; ycfM*	Formation of capsule and its lipopolysaccharides (LPS).	[264]
*wbbY; wbbZ*	Modify LPS composition.	[267]
*entS*	Production of enterobactin.	[240,269]
*armA; aacA4*; *aacC2*; *aadA1; aac(6’)-Ib*	Aminoglycosides resistance.	[246,247]
*bla*_KPC-2_; *bla*_KPC-3_	Carbapenem, clavulanic acid and tazobactam resistance.	[249,250,251]
*acrAB*, *qnrB; qnrS*	Quinolones resistance.	[246,254]
*bla_SHV_; bla_TEM_; bla_CTX-M_*	Carbapenems resistance.	[254]
*lpxM*	Polymyxin resistance.	[246]
*ramR*; *rpsJ*; *tetA*	Tigecycline resistance.	[255]

**Table 4:** Examples of virulence and drug resistance-related genes reported for *Acinetobacter baumannii*.

Genes	Product Function	Reference
*ompA*	Encodes OmpA protein, involved in the adhesion of epithelial cells and plays essential roles in the regulation of aggressiveness and biofilm formation.	[305,306]
*csu; bap*	Encodes Csu pili and biofilm-associated proteins that promote adherence to skin epithelial cells during initial stage of the colonization process.	[18,313]
*zigA*	Metal elimination system essential for its metabolism.	[318,319]
*bla_ctx-m_*; *bla_ges_*; *bla_per_*; *bla_sco_*; *bla_shv_*; *bla_tem_*; *bla_veb_*	Penicillin and cephalosporin (except cephamycin) resistance.	[280,281,282,283]
*katG*	Hydrogen peroxide resistance.	[284]
*tetA*; *tetB; tetM*	Tetracyclines, minocycline and doxycycline resistance.	[18]
*gyrA*; *parC*	Fluoroquinolones resistance.	[286]
*aac(3′)-Ia*; *ant(2’)-Ia*; *ant(3″); armA*; *rmtA*; *rmtB*; *rmtC*; *rmtD*	Aminoglycosides resistance.	[18,288,289]
*adeABC* and adeM	Efflux pumps (gentamicin resistance)	[290]
*pmrC; pmrA*; *prmB; lpsB*; *lptD*; *vacJ*	Polymyxins resistance.	[291,292]
*oxa_-23_*; *oxa_-51_*	Carbapenems resistance.	[293]

**Table 5:** Examples of virulence and drug resistance-related genes reported for *Pseudomonas aeruginosa*.

Genes	Product Function	Reference
*exoS; exoT; exoY; exoU*	Encode ExoS, ExoT, ExoY and ExoU proteins.	[323,339]
*phzI*; *phzII*; *phzH*; *phzM*; *phzS*; *plcH*a; *plcN*	Products are elastase and alkaline protease.	[357]
*pilA*; *pilB*	Expression of pili; participates in bacterial adhesion and the colonization of epithelial surfaces.	[357]
*oxA*	Exotoxin A; contributes to tissue damage in the early stages of infection, in addition to the uptake of important nutrients for its growth.	[357]
*OprD*	Carbapenems resistance.	[325]
*mexAB-oprM; mexXY-(oprA); mexCD-oprJ*; *mexEF-oprN*	Multi-drug resistance.	[326]
*gyrA*; *gyrB*; *parC*; *parE*	Fluoroquinolones resistance.	[327]
*mcr-1*; *bl;_M-1_*	Polymyxins resistance.	[330,331]
*exoS*; *exoU*	Multi-drug resistance.	[339]

**Table 6:** Examples of drug resistance-related genes reported for *Enterobacter* sp.

Genes	Product Function	References
*bla* *_TEM-1_*	Multi-drug resistance.	[22]
*bla_CTX-M_*	Cephalosporins resistance.	[220]
*bla_IMP-8_; bla_CTX-M-3_; qnrS1; bla_CTX-M-14_; bla_TEM-1B_; bla_OXA-1_; catB3; sul1*	Multi-drug resistance—aminoglycosides, β-lactams, fluoroquinolones, fosfomycin, macrolides, phenicols, rifampicin and sulfonamides.	[371]
*bla_NDM_; bla_VIM_; bla_IMP_*	Multi-drug resistance—carbapenems, cefoperazone, sulbactam, trimethoprim, sulfamethoxazole, aminoglycosides (gentamicin and amikacin) and fluoroquinolones (ciprofloxacin).	[374]
*bla_KPC-2_*, *bla_KPC-3_*, *bla_KPC-4_* and *bla_NDM-1_*	Carbapenems resistance.	[375]

## Data Availability

Not applicable.
